# Lipid catabolism inhibition sensitizes prostate cancer cells to antiandrogen blockade

**DOI:** 10.18632/oncotarget.17359

**Published:** 2017-04-21

**Authors:** Thomas W. Flaig, Maren Salzmann-Sullivan, Lih-Jen Su, Zhiyong Zhang, Molishree Joshi, Miguel A. Gijón, Jihye Kim, John J. Arcaroli, Adrie Van Bokhoven, M. Scott Lucia, Francisco G. La Rosa, Isabel R. Schlaepfer

**Affiliations:** ^1^ Division of Medical Oncology, Department of Medicine, University of Colorado, Anschutz Medical Campus, Aurora, Colorado, USA; ^2^ Department of Pharmacology, University of Colorado, Anschutz Medical Campus, Aurora, Colorado, USA; ^3^ Department of Pathology, University of Colorado, Anschutz Medical Campus, Aurora, Colorado, USA

**Keywords:** CPT1A, prostate cancer, enzalutamide, ranolazine, INPP5K

## Abstract

Prostate cancer (PCa) is the most common malignancy among Western men and the second leading-cause of cancer related deaths. For men who develop metastatic castration resistant PCa (mCRPC), survival is limited, making the identification of novel therapies for mCRPC critical. We have found that deficient lipid oxidation via carnitine palmitoyltransferase (CPT1) results in decreased growth and invasion, underscoring the role of lipid oxidation to fuel PCa growth. Using immunohistochemistry we have found that the CPT1A isoform is abundant in PCa compared to benign tissue (n=39, p<0.001) especially in those with high-grade tumors. Since lipid oxidation is stimulated by androgens, we have evaluated the synergistic effects of combining CPT1A inhibition and anti-androgen therapy. Mechanistically, we have found that decreased CPT1A expression is associated with decreased AKT content and activation, likely driven by a breakdown of membrane phospholipids and activation of the INPP5K phosphatase. This results in increased androgen receptor (AR) action and increased sensitivity to the anti-androgen enzalutamide. To better understand the clinical implications of these findings, we have evaluated fat oxidation inhibitors (etomoxir, ranolazine and perhexiline) in combination with enzalutamide in PCa cell models. We have observed a robust growth inhibitory effect of the combinations, including in enzalutamide-resistant cells and mouse TRAMPC1 cells, a more neuroendocrine PCa model. Lastly, using a xenograft mouse model, we have observed decreased tumor growth with a systemic combination treatment of enzalutamide and ranolazine. In conclusion, our results show that improved anti-cancer efficacy can be achieved by co-targeting the AR axis and fat oxidation via CPT1A, which may have clinical implications, especially in the mCRPC setting.

## INTRODUCTION

Prostate cancer (PCa) is the most commonly diagnosed malignancy and the second highest contributor to cancer deaths in men in the Western World [1]. Currently, the standard approach to first-line systemic treatment for advanced PCa is built on androgen deprivation therapy (ADT) and although nearly all patients initially respond, resistance to ADT develops overtime, frequently related to restored AR signaling [2]. Since patients with metastatic, castration-resistant prostate cancer (mCPRC) have a limited survival, improved treatment options in this setting are critical. The role of lipid use in mCRPC is incompletely understood, but it may involve a gene expression program orchestrated by novel and restored AR signaling pathways [3]. In fact, lipid synthesis is a major target of androgen action in PCa cells [4, 5] but the identification of lipogenic enzymes as targets for therapy remains challenging.

Although new Highly-Effective Androgen Therapy (HEAT) such as the androgen receptor inhibitor Enzalutamide [6] and the androgen biosynthesis inhibitor Abiraterone acetate [7] have been shown to provide a survival advantage in mCRPC, virtually all patients acquire secondary resistance [8]. In some cases, this is likely mediated by AR variants that promote androgen-regulated programs [9], which also affects the lipid metabolic program [10]. Some AR variants are constitutively active isoforms of the AR lacking the ligand-binding domain, yet retaining transcriptional activity in a ligand-independent fashion. Of these, AR variant-7 (ARv7) has been identified as the most relevant in insensitivity to HEAT in men with advanced PCa [8].

The role and relative contribution of lipids in PCa appears different from other cancer types. While most cancer cells primarily utilize glycolysis, several lines of evidence suggest that PCa utilizes lipid as fuel to a greater extent, although the mechanisms are poorly understood [11, 12]. The overexpression of key lipid enzymes in PCa is characteristic of both primary and advanced disease [13], suggesting that targeting lipid catabolism in PCa may be more relevant than in other cancer types. In particular, our studies focus on the CPT1A enzyme and its role in PCa, facilitating the entry of long chain fatty acids into the mitochondria for oxidation [12, 14] and supporting the importance of this lipid utilization pathway in PCa. Several lines of evidence indicate that intracellular lipid oxidation is important in cancer cell survival [15], resistance to radiation [16], oxidative stress [17] and activation of oncogenic signaling pathways [18]. Altogether, lipid oxidation is an important component of metabolic reprograming in cancer that remains to be exploited for therapy in PCa.

One way to study the role of lipid oxidation involves the use of clinically-approved metabolic inhibitors that could facilitate translational studies [19]. Ranolazine is a partial beta-oxidation inhibitor that is FDA-approved for the treatment of angina and reduces beta oxidation in the heart [20]. Another inhibitor is Perhexiline [21], which works by inhibiting CPT1 specifically, but unlike etomoxir [22], it is clinically used outside of the USA (Pexsig, Australia). Etomoxir is not FDA approved. Presently, there are no studies of these metabolic drugs in combination with anti-androgens, making them attractive tools to explore the role of lipid oxidation in PCa and design novel optimal therapies.

In our previous studies [14], we used an acute dose of etomoxir that resulted in cell death by apoptosis and a dramatic downregulation of the AR mRNA expression. In this manuscript, we have focused on a CPT1A knockdown (KD) model, which represents a long-term adaptation of the LNCaP cells to chronic downregulation of the CPT1A enzyme. We have found that by maintaining AR expression and action, the CPT1AKD cells are able to survive (albeit slower growth). Whether the acute effect of etomoxir on AR mRNA is direct, mediated by CPT1 inhibition or caused by off-target effects of the drug remains unknown. In fact, etomoxir can also work as a direct ligand for the peroxisome proliferator-activated receptor-α (PPARα) [23], suggesting that off-target effects of etomoxir may also contribute to its effects on AR and cell viability.

In this study, we have evaluated the synergistic effects of combining CPT1A inhibition and anti-androgen therapy in PCa cells to better understand the relationship of these pathways. We first examined the phenotypic changes associated with CPT1AKD and knockout (KO) to validate the relevance of this pathway in PCa and once the importance was established, we evaluated the synergistic effects with antiandrogens. We have found that fat oxidation blockade via CPT1AKD results in a compensatory increase in AR pathway activation leading to an increased sensitivity to enzalutamide (MDV3100). Furthermore, pharmacological inhibition of beta-oxidation also synergized with enzalutamide in decreasing PCa growth, highlighting the unexplored crosstalk between the AR axis and fat oxidation in advanced PCa.

## RESULTS

### CPT1A is increased in advanced prostate cancer

**Figure 1:**
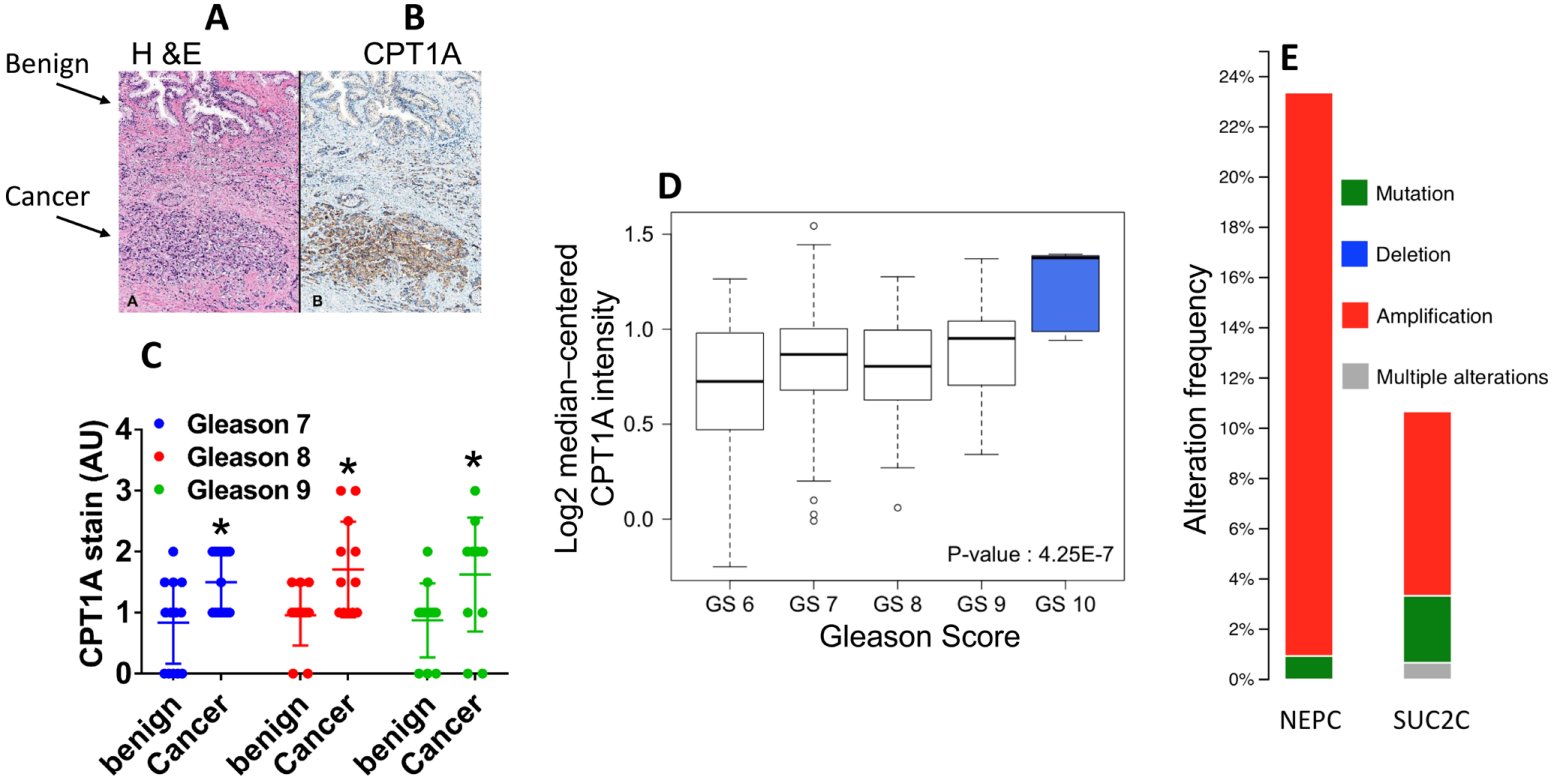
CPT1A expression is increased in advanced prostate cancer **A.**-**B.** Representative images of serial sections of benign and cancer tissue (arrows) from the same RRP specimen stained with H&E (A) or CPT1A (B) specific stain. **C.** Quantification of CPT1A stain in 39 RRP specimens grouped by Gleason Score (GS). ANOVA: p < 0.0001, post hoc tests for each GS group compared to benign: GS7, n = 15, *p = 0.02; GS8, n = 12 *p = 0.018; GS9, n = 12, *p = 0.02. AU = arbitrary units. **D.** Graphical representation of Oncomine data (Setlur dataset) showing increased expression of CPT1A with advanced Gleason score. **E.** Graph from cBioPortal showing CPT1A gene amplification in neuroendocrine (NEPC) and adenocarcinoma (SUC2C) samples in 2 recent datasets (Trento /Cornell/Broad 2016 and stand-up-2-cancer/PCF projects, respectively).

In order to examine the levels of CPT1A expression in human PCa we evaluated 39 cancer prostatectomy cases from University of Colorado Hospital, Figure 1. Serial sections of whole-mount prostate samples were used to identify prostate cancer by H & E staining followed by CPT1A specific staining (Figure 1A). The expression of CPT1A was cytoplasmic and only observable in the epithelial component of the luminal glands, with no staining in the stroma or basal cell layer, Figure 1B and Supplementary Figure 1 (additional 40X images). Increased expression was observed in the involved cancerous areas, especially the higher grade areas (Gleason patterns 4 and 5). Figure 1C shows the results of the CPT1A stain analysis in 39 whole prostatectomies by Gleason score. These results are in agreement with the database from Oncomine™ as shown in Supplementary Table 1 and Figure 1D, where increased expression of CPT1A was observed in high-grade PCa [24]. We have also examined c-BioPortal to assess gene alterations. We have found CPT1A to be amplified in 22 % of cases (*n* = 107) in the neuro-endocrine prostate cancer (NEPC, Figure 1E) dataset from the Trento/Cornell/Broad 2016 database [25], which also brings attention to the drug-resistant PCa tumors, including LNCaP cells that were treated for a long time with enzalutamide. The fact that they find 22% of their cases with CPT1A amplification underscores the potential relevance of a metabolic treatment for high-risk neuro-endocrine-type and castration-resistant PCa. Additionally, another important dataset from the stand-up-2-cancer group (SU2C/PCF Dream team, Figure 1E) also shows CPT1A gene altered (mainly amplification) in 11 % of PCa cases (*n* = 150) [26]. Additional database studies are shown in Supplementary Figure 1.

### CPT1A is needed to maintain viability and invasion of prostate cancer cell lines

**Figure 2:**
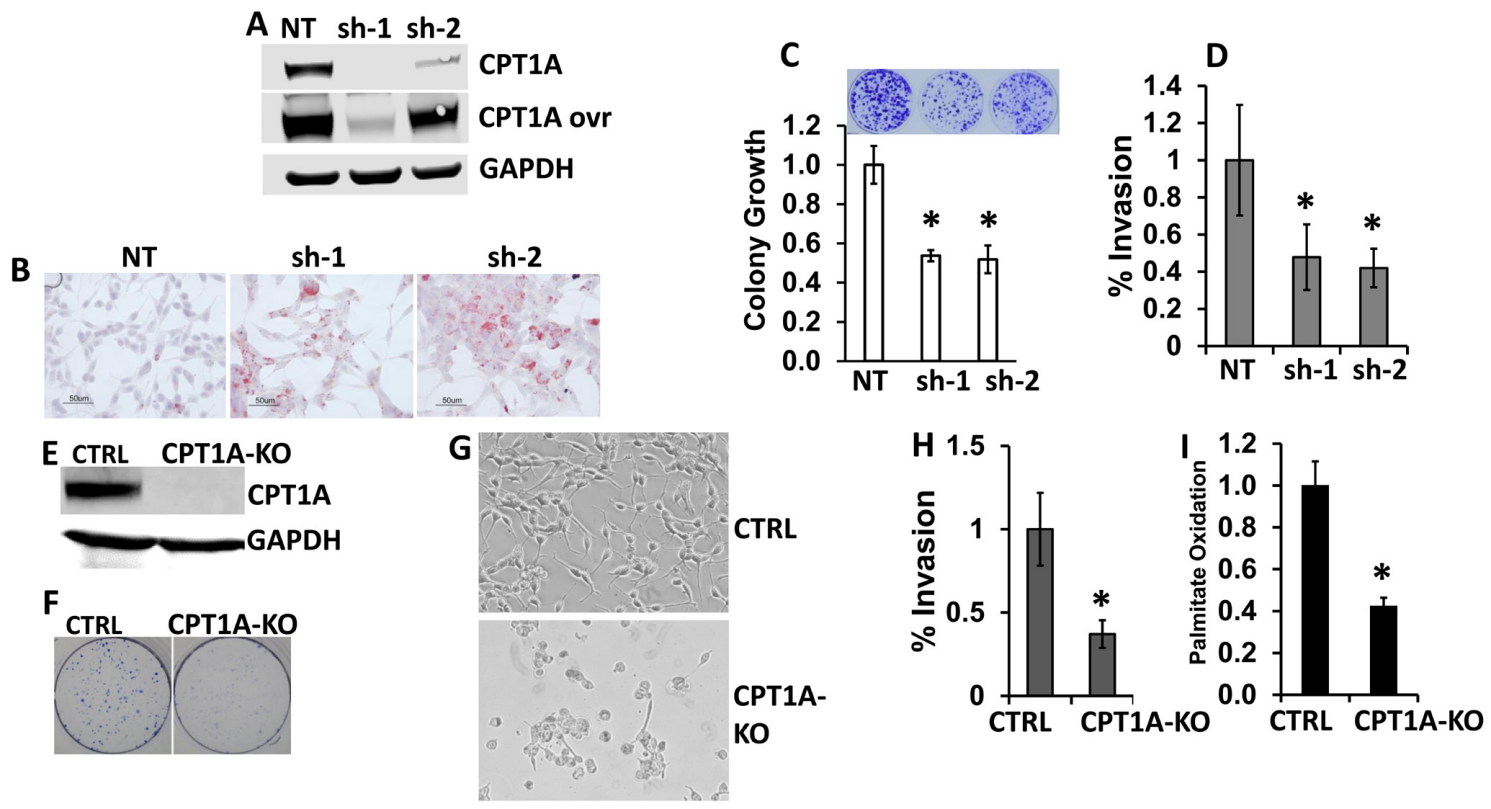
CPT1A is needed to maintain viability and invasion of prostate cancer cell lines **A.** Western blot of CPT1A- knockdown (KD) LNCaP clones (sh-1 and sh-2). **B.** Increased accumulation of lipid in CPT1A-KD clones. Red stain shows lipid droplets. **C.** Decreased clonogenicity of CPT1A-KD clones, *p < 0.01 compared to control (NT). **D.** Decreased invasion of KD clones compared to controls (NT), *p < 0.01. **E.** Western blot of CPT1A-KO CRISPR-edited LNCaP cells (CPT1A-KO). **F.**-**G.** Representative clonogenic assay (F) and live cell photographs (G) of CPT1A-KO cells. **H.** Decreased invasion of CPT1A-KO cells compared to controls (CTRL), *p < 0.01. **I.** Decreased palmitate oxidation in CPT1A-KO cells compared to control clones, *p < 0.01.

We have previously shown that knockdown (KD) of CPT1A decreases beta-oxidation in LNCaP cells [12, 14]. In this work, we further examined biochemical and growth characteristics in these KD cells, Figure 2. We found increased lipid droplet accumulation and decreased clonogenic growth and invasion in the CPT1A-KD (sh-1, -2) clones compared to controls (NT, non-targeting shRNA), Figure 2B-2C-2D. Furthermore, CRISPR editing of CPT1A gene in LNCaP cells also mirrored the effects of the KD clones, but with a more pronounced phenotypic change, leading to markedly reduced cell viability and death, Figure 2E-2F-2G. Additionally, these knockout (KO) cells also show decreased invasion potential and decreased lipid oxidation compared to controls, Figure 2H-2I. The lack of obliteration of lipid oxidation in the KO cells is likely due to peroxisome oxidation, since these organelles also oxidize lipids. The CPT1A-KO results are in agreement with the embryonic lethality seen in Cpt1a-KO mice, underscoring a critical role in survival for CPT1A [22]. Since the CPT1A-KO cells exhibited substantial reduced viability, we proceeded with shRNA-derived clones for the subsequent studies.

### Increased AR action and AR-regulated genes in CPT1A-KD LNCaP cells

**Figure 3:**
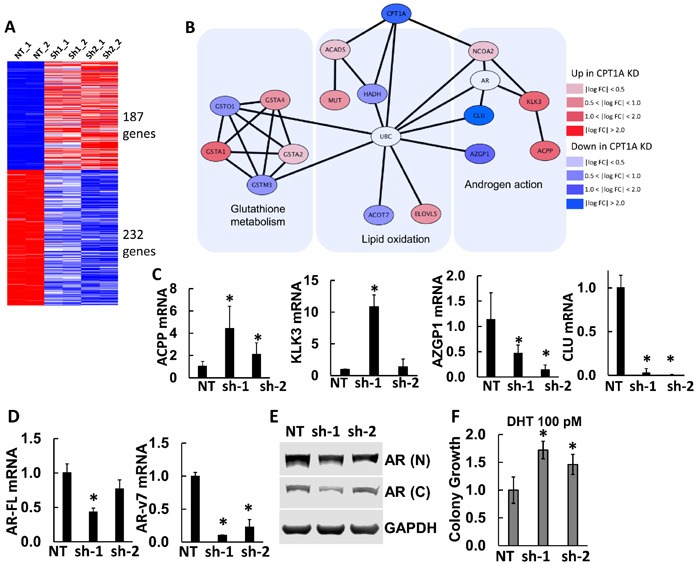
Gene expression results from CPT1A-KD LNCaP cells. A Results from RNAseq analysis of CPT1AKD cells compared to controls (NT). Upregulated (red) and downregulated (blue) genes were used to generate a protein interaction network. **B.** Protein interaction diagram of the significant genes from (A). The figure is drawn using the STRING database through Cytoscape (http://cytoscape.org) to identify interactions of the up- or down-regulated genes from CPT1A knockdown. AR and UBC genes have a non-colored background because they are not significantly changed in the RNAseq analysis. **C.** Semi-quantitative RTPCR of significant AR-regulated genes: ACPP (acid phosphatase, *p ≤ 0.01) KLK3 (PSA, *p < 0.001), AZGP1 (*p ≤ 0.01) and CLU (*p ≤ 0.001). **D.** RTPCR of AR-full length (AR-FL, *p < 0.01), AR variant 7 (AR-v7, *p ≤ 0.001) compared to NT controls. **E.** Representative western blot of full length AR in CPT1AKD clones. N = N-terminus specific antibody (N-20). C = C-terminus specific antibody (C-19). **F.** Clonogenic assay of CPT1AKD clones in the presence of DHT (100 pM), ANOVA p = 0.006, *p ≤ 0.04 compared to NT control.

In order to identify the molecular basis for the increased lipid accumulation and decreased growth in the LNCaP CPT1A-KD cells, we performed RNAseq studies. Figure 3A-3C show results of our gene expression analysis. There were 187 and 232 common significant up and downregulated genes respectively (see Supplementary Table 4 for a complete list of significant genes, pathway analysis and String Network). Interestingly, pathway analysis only identified 2 significant pathways (glutathione metabolism and cell adhesion), which likely reflects the decreased growth due to oxidative stress and increased cell adhesion (less invasion) with decreased CPT1A expression, Figure 2C-2D. The STRING interaction analysis, however, pointed to AR-regulated genes like KLK3 (PSA), ACPP, CLU and AZGP1 in the proximity of CPT1A. Thus, using the STRING database through the Cytoscape software (http://cytoscape.org/, ver. 3.4) we identified AR-regulated genes associated with decreased CPT1A expression, Figure 3B. We verified these genes by RTPCR as well as AR full length (AR-FL) and AR variant 7 (ARv7, lacking the ligand-binding domain), Figure 3C-3D. As in our previous publication with the CPT1A inhibitor etomoxir [14], we found a significant decrease in ARv7 mRNA expression in both clones, but only significant changes in AR-FL mRNA with the sh1 clone. These results were mirrored by the AR protein expression, although a significant amount of AR was still present in both clones, Figure 3E. Lastly, clonogenic assays in presence of dihydrotestosterone (DHT 100 pM) resulted in a significant boost in colony number, suggesting increased AR-FL action in the CPT1AKD clones compared to control, Figure 3F.

### Decreased phosphatidylinositol (PI) and increased INPP5K expression are associated with decreased activation of AKT and increased expression of AR in CPT1A KD cells

**Figure 4:**
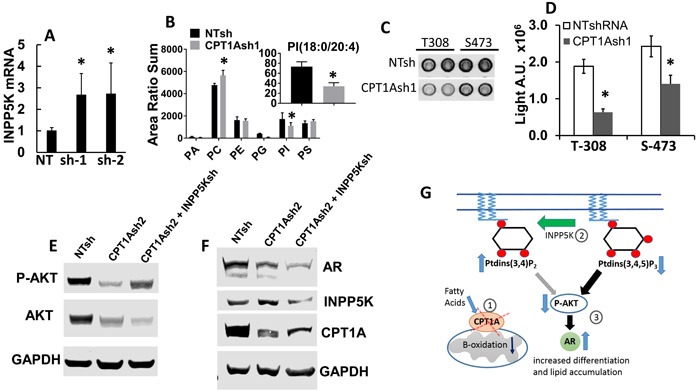
Decreased phosphatidylinositol (PI) and increased INPP5K expression result in decreased activation of AKT and increased expression of AR in CPT1A KD cells **A.** Increased mRNA of INPP5K (inositol phosphatase, *p < 0.01) in LNCaP CPT1A KD cells. **B.** Phospholipid analysis of the CPT1AKD cells. The x-axis represents phosphatidic acid, phosphatidylcholine, phosphatidylethanolamine, phosphatidylglycerol, phosphatidylinositol and phosphatidylserine, respectively. The graph represents the sum of the area ratios of every molecular species in each phospholipid class, normalized to the total phospholipid content in each extract of cells, and it shows decreased total abundance of PI species and increased PC species (*p ≤ 0.05 compared to NT control). The inset represents the area ratio of specific PI(18:0/20:4) species (*p < 0.05 compared to NT control). **C.** Representative array blot (Cell Signaling) of CPT1A-KD cells showing decreased p-AKT signal. **D.** Signal array quantification, *p < 0.001 compared to control (NT clones). **E.**-**F.** Representative western blots showing CPT1A-KD and double CPT1A+INPP5K KD cell lysates probed for p-AKT, INPP5K, CPT1A and AR in the double KD cell lysates. **G.** Diagram of the site of action of INPP5K, PI and its potential effect on AR expression via decreased activation of p-AKT. The decreased expression of CPT1A (1) leads to increased phospholipid degradation and increased INPP5K phosphatase activity, (2) resulting in decreased AKT expression and activation (3). This consequent decrease in p-AKT leads to increased AR protein expression and action, increasing PSA (KLK3) and resulting in a more differentiated phenotype.

Since the CPT1A KD cells appear to have a more differentiated phenotype based on increased PSA and lipid accumulation, we searched our RNAseq data for genes related to lipids and AKT activation. INPP5K codes for an inositol phosphatase that is able to de-phosphorylate phosphatidylinositols (PI) involved in AKT activation, like phosphatidylinositol 3,4,5-triphosphate or PI(3,4,5)P3, at the plasma membrane [27]. Furthermore, AR content is known to be regulated by phosphorylation via AKT [28], and a reciprocal feedback regulation of AKT and AR signaling has been observed in PCa [29], via the PHLPP phosphatase. Thus, we examined the expression of INPP5K, total and (18:0/20:4)-specific PI content, phospho-AKT and AR in the LNCaP CPT1A-KD cells compared to controls. Figure 4 shows increased expression of INPP5K, concomitant with decreased content of PI and decreased p-AKT in CPT1A-KD cells, Figure 4A-4B-4C-4D. We found that while the levels of other phospholipid classes, particularly the abundant phosphatidylcholines (PC) and phosphatidylethanolamines (PE) do not decrease in the CPT1Ash1 cells, PI species decrease significantly, in particular PI(18:0/20:4) (Figure 4B, inset), which are the precursors of phosphorylated species that have been shown to activate AKT [30], and are likely best targets for INPP5K activity [31]. Moreover, downregulation with INPP5K-specific shRNA in the CPT1A-sh2 cells resulted in recovery of AKT activation (S473), concomitantly with a reduction in AR expression, Figure 4E-4F. Interestingly, downregulation of INPP5K by itself in LNCaP cells did not increase pAKT or change CPT1A expression, (Supplementary Figure 3) suggesting a CPT1A role in mediating these effects. A diagram depicting the potential role of INPP5K and PI regulating AR content via AKT is shown in Figure 4G.

### Combinatorial effects of CPT1A inhibition and anti-androgen therapy in human PCa cells

**Figure 5:**
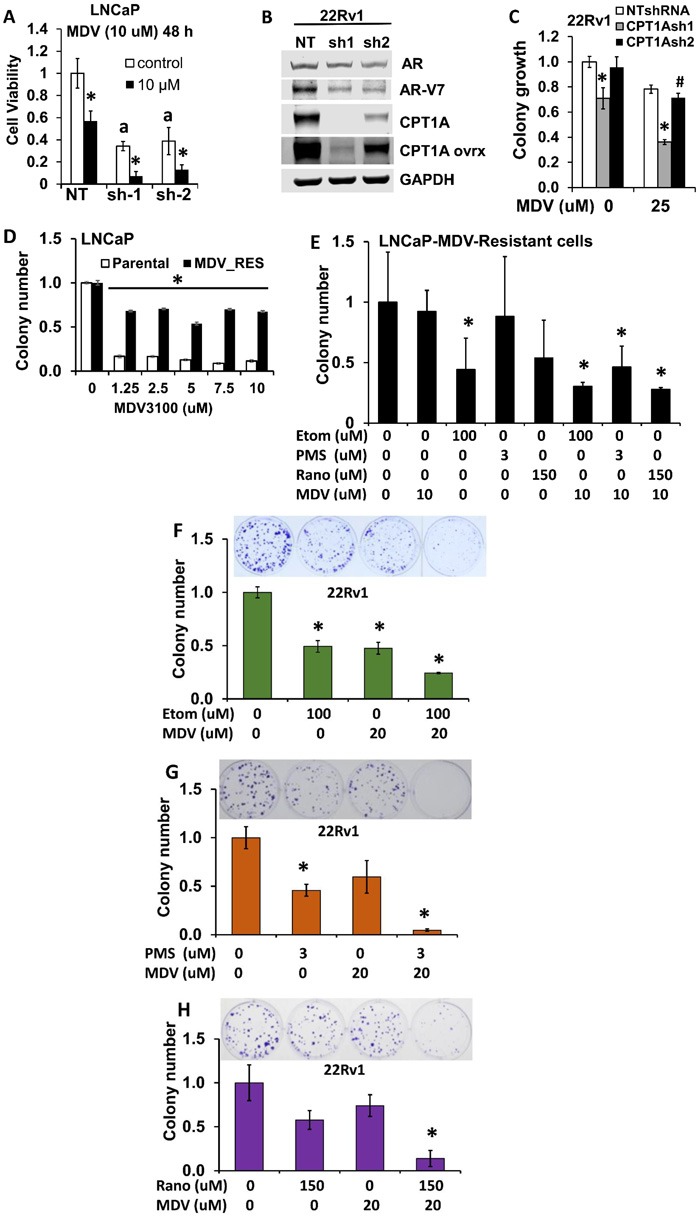
Combinatorial effects of CPT1A inhibition and anti-androgen therapy in human PCa cells **A.** CPT1A-KD cells show increased sensitivity to the anti-androgen enzalutamide (MDV) at 48 hours, a p < 0.001, *p < 0.01. **B.** 22RV1 CPT1A-KD cells show decreased protein expression of the ligand-independent AR variant 7 (ARv7). **C.** 22Rv1 cells deficient in CPT1A show decreased growth and increased sensitivity to enzalutamide, ANOVA, p < 0.001. Post hoc Tukey tests: *p < 0.001 compared to control clone (NTshRNA) and vehicle treatment, #p = 0.035 compared to vehicle treatment. **D.** LNCaP-enzalutamide resistant cells can grow in the presence of enzalutamide (MDV), ANOVA for MDV-resistant cells p < 0.001, Post hoc *p < 0.001 compared to parental cell line for each drug dose. **E.** Increased sensitivity to the combination of ranolazine (Rano), etomoxir (Etom) or perhexiline (PMS) with enzalutamide (MDV) in LNCaP-enzalutamide resistant cells, post hoc tests *p < 0.05 compared to vehicle. **F.**-**H.** Increased sensitivity to the combination of etomoxir (F), perhexiline (G) or ranolazine (H) with enzalutamide in 22Rv1 cells, post hoc tests *p < 0.001 compared to vehicle treatments.

Since we observed increased expression of AR-regulated genes (Figure 3C) and increased sensitivity of the CPT1AKD cells to androgens (Figure 3F), we next studied the sensitivity of the cells to anti-androgens. LNCaP CPT1A-KD and control cells treated with enzalutamide (MDV) were examined for viability after 48 hours by trypan exclusion assay, Figure 5. The LNCaP CPT1A-KD cells were more sensitive to the enzalutamide treatment (2.8-fold for NT control vs. 6-fold for the Sh-1 KD clone compared to their respective vehicle treatments), Figure 5A. Furthermore, when we decreased CPT1A in the 22Rv1 cell line with the same shRNA’s as for the LNCaP cells, Figure 5B, we also observed increased sensitivity to enzalutamide, albeit to a lesser extent than in the LNCaP clones, Figure 5C. Particularly, we observed decreased growth in the clone with lowest CPT1A expression (sh-1) and addition of enzalutamide resulted in a further 50% (*p* < 0.001) decrease in growth. The sh-2 clone with a lesser decrease in CPT1A expression showed no change in growth compared to control, but a 30% (*P* < 0.01) decrease in growth was observed with enzalutamide. The expression of full length AR was not significantly changed in the CPT1AKD 22Rv1 cells but we did observe a substantial decrease in ARv7 expression, Figure 5B, thus increasing the AR full length to AR variant ratio, and likely enhancing the sensitivity to enzalutamide, like previously observed with the LNCaP model, Figure 3.

To better explore the potential clinical application of this work, we next examined the effects of combining fat oxidation inhibitors and enzalutamide on PCa growth. Enzalutamide-Resistant LNCaP cells and LNCaP parental cells (Figure 5D) were used to study clonogenic growth with enzalutamide in combination with etomoxir, perhexiline or ranolazine, Figure 5E. Significant decreases in colony growth were observed in the combination treatments, as well as with the single treatment with etomoxir. Since 22Rv1 cells are another model of increased AR variant content and intrinsic resistance to enzalutamide, we treated 22Rv1 cells with etomoxir, perhexiline and ranolazine alone and in combination with enzalutamide (MDV), Figure 5F-5G-5H respectively. The combination treatments resulted in a significant decrease of 22Rv1 growth (about 10-fold, *p < 0.001 compared to vehicle treatment). To test for the synergy of the drug combinations, we performed MTS assays with 22Rv1, LNCaP-MDV resistant and TRAMPC1 cells and used the CalcuSyn program to estimate the combinatorial index (CI). Table 1 shows the affected fractions and CI of all the combinations tested in our cell models using MTS proliferations assays and 8 replicates per condition. For reference, CI = 1 indicates additive value of a combination, while CI < 1 indicates synergy. Interestingly, the CI values not only show synergy with combinatorial drug doses used for the clonogenic assays, but also for lower drug concentrations. One exception to these observations was the MDV+etomoxir combination in TRAMPC1 cells, where antagonism was observed with the lowest doses (CI = 1.5).

**Table 1:** Combinatorial Index for the drug combinations

**22Rv1 cells**			
**MDV3100 (uM)**	**Etomoxir (uM)**	**Fa**	**CI**
10	50	0.131	0.639
20	100	0.463	0.893
30	150	0.528	1.272
**MDV3100 (uM)**	**Perhexiline (uM)**	**Fa**	**CI**
10	1	0.190	0.497
20	3	0.428	0.848
30	6	0.601	1.167
**MDV3100 (uM)**	**Ranolazine (uM)**	**Fa**	**CI**
10	100	0.123	0.702
20	150	0.429	0.941
30	200	0.520	1.286
**LNCaP-MDV-Resistant cells**			
**MDV3100 (uM)**	**Etomoxir (uM)**	**Fa**	**CI**
5	50	0.227	0.556
10	100	0.491	0.859
20	150	0.630	1.351
**MDV3100 (uM)**	**Perhexiline (uM)**	**Fa**	**CI**
5	1	0.353	0.342
10	3	0.613	0.572
20	6	0.701	1.035
**MDV3100 (uM)**	**Ranolazine (uM)**	**Fa**	**CI**
5	100	0.411	0.642
10	150	0.499	1.023
20	200	0.648	1.499
**TRAMPC1 cells**			
**MDV3100 (uM)**	**Etomoxir (uM)**	**Fa**	**CI**
1	50	0.407	1.523
3	75	0.576	0.336
6	150	0.696	0.289
**MDV3100 (uM)**	**Perhexiline (uM)**	**Fa**	**CI**
1	1	0.365	0.421
3	3	0.779	0.512
6	6	0.891	0.710
**MDV3100 (uM)**	**Ranolazine (uM)**	**Fa**	**CI**
1	25	0.234	0.624
3	50	0.756	0.670
6	100	0.791	1.261

Fa = affected fraction

CI = combinatorial index

The combinations used for the clonogenic assays are indicated in grey shade.

### Combinatorial effects of beta-oxidation inhibition and anti-androgen therapy in mouse TRAMPC1 cells and human 22Rv1 xenografts

**Figure 6:**
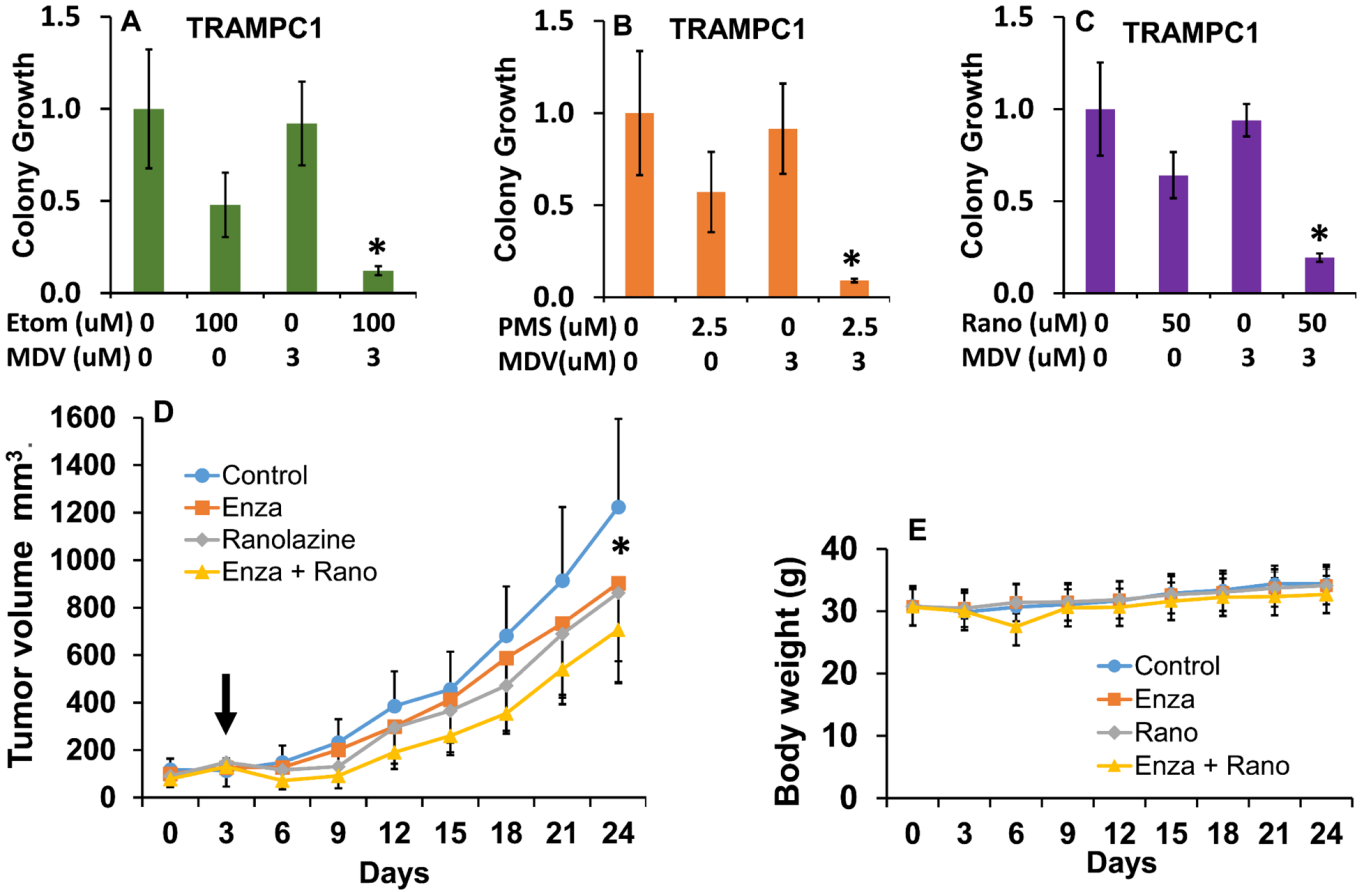
Combinatorial effects of beta-oxidation inhibition and anti-androgen therapy in mouse TRAMPC1 cells and human 22Rv1 xenografts **A.**-**C.** Clonogenic assay showing the effects of the combination of ranolazine (A), etomoxir (B) or perhexiline (C) with enzalutamide (MDV) in mouse TRAMPC1 cells, post hoc tests *p ≤ 0.03 compared to individual treatments. **D.** Tumor growth in mice treated with vehicle (0.5% methyl cellulose with 0.1% tween 80), enzalutamide (20 mg/kg), ranolazine (40 mg/kg) or a drug combination (enza+rano) via gavage over 21 days. Five mice with 2 tumors each were used for each treatment group. Arrow indicates beginning of treatment. Repeated measures ANOVA showed significant changes due to the effect of treatment over time (p < 0.001, F = 2.5 df = 24). *p < 0.05 (paired t-test comparing control to combination group). **E.** Effect of treatments on mouse body weights. No significant changes and no signs of toxicity were observed throughout the study.

The murine TRAMPC1 cells are a prostate cancer cell model that expresses AR [32] and has been recently used for enzalutamide studies [33]. As shown in Figure 6, the TRAMPC1 cells are significantly sensitive to the combination of fat burning inhibitors and enzalutamide, expanding our results beyond human cell lines. Interestingly, the effect of the metabolic inhibitors alone reduced the growth of TRAMPC1 cells *in vitro* by nearly 50% for the 3 drugs, and the addition of enzalutamide resulted in a strong synergistic inhibition of growth. Table 1 contains the combinatorial index calculations in the TRAMPC1 cells.

To test our drug combination results in a mouse model, we generated 22Rv1 mouse xenografts in nude mice and treated them systemically with vehicle, enzalutamide, ranolazine or a combination of both (enza + rano) over 3 weeks. Figure 6D-6E shows the treatment effects. We used low doses of the drugs to maximize the combinatorial effects and minimize toxicity. Interestingly, the ranolazine treatment alone showed a decrease in growth similar to enzalutamide, but only the combination treatment produced a significant change compared to control treatment.

## DISCUSSION

The results of our study point to an important role for CPT1A in anti-androgen resistance. Although CPT1A is not an oncogene, it has a critical role in PCa in light of the addiction of PCa to beta-oxidation. Furthermore, cells with CPT1A KO via CRISPR editing exhibited a marked reduction in viability, whereas in the case of CPT1A shRNA clones the cells remained viable, suggesting the importance of minimal CPT1A protein expression in the context of PCa survival. In fact, CPT1A is also implicated in the invasion capacity of the cells (Figure 2), which could be explained by decreased energy production from beta-oxidation [14], and/or increased cell adhesion via NCAM2 and CADM1 expression (Supplementary Table 4). Overall, these results suggest that additional functions of CPT1A remain to be explored beyond its rate-limiting step function in mitochondrial beta-oxidation [34]. The identification of CPT1A in Gleason 10 and its high level of alteration in the NEPC dataset suggest that CPT1A may have additional benefit as a biomarker for high-risk or neuroendocrine-type cancers [25, 35] .

The rational for combining anti-androgen therapy and lipid oxidation inhibitors stems from the fact that AR is known to stimulate aerobic glycolysis, lipid oxidation and several anabolic processes in PCa [13, 36, 37]. Thus, it is possible to conceive that upregulation of AR action is a compensatory response to chronic lipid oxidation blockade. In this study, we have found that inhibition of CPT1A increases AR action reflected in the increased expression of AR-regulated genes (e.g. PSA and CLU) and increased sensitivity to DHT and enzalutamide, Figures 3 and 5. These effects occur in the context of decreased ARv7 expression, which is associated with a high level of resistance to HEAT, suggesting that increasing the ratio of full length AR (target of enzalutamide) to ARv7 is important for increasing the sensitivity to enzalutamide. In fact, our 22Rv1 model with decreased CPT1A expression showed decreased ARv7 expression and increased sensitivity to enzalutamide without change in full length AR. This is in agreement with other studies suggesting that full length AR is the driver of enzalutamide resistance in LNCaP cells, while AR variants might have the dominant role in 22Rv1 cells [38].

The mechanism of decreased CPT1A protein resulting in AR expression changes is not fully elucidated, but we have found that it is associated with decreased AKT activation. This may be driven by a breakdown of plasma membrane phospholipids in order to compensate for the defect in beta-oxidation, but resulting in less AKT anchoring to the membrane for activation. This regulation of AKT signaling by fat oxidation has been recently described in normal epidermis [39] and hematopoietic cells [40], where AKT overexpression is able to suppress CPT1A, suggesting a novel modulatory loop between AKT and CPT1A for future study. From our RNAseq results, although not significantly changed in both clones, we have identified INPP5K (also known as SKIP) as a potential phosphatase mediating the decreased AKT activation in LNCaP cells. In fact, a previous study [29] has already reported about the reciprocal negative regulation between AR and AKT signaling, *via* another phosphatase named PHLPP. The role of INPP5K in PCa is currently unexplored, but our data suggests that it likely functions as a regulator of phosphatidylinositol (PI) substrate availability at the membrane, influencing the activation of AKT [41]. In fact, Oncomine results show decreased expression of INPP5K in PCa compared to normal tissue (Supplementary Figure 3), suggesting a possible tumor suppressor activity of INPP5K [42].

Additionally, we have shown that the decrease in p-AKT can be reversed by reducing INPP5K expression, resulting in a decrease of AR content. These results are in agreement with recent studies showing a compensatory crosstalk between PI3K-AKT-AR pathways in PCa [43]. In another study [44], pharmacological inhibition of AKT resulted in increased expression of AR content and AR-dependent genes, an effect that was overcome by addition of the anti-androgen bicalutamide. Altogether these studies support our observations that decreased CPT1A expression results in decreased AKT activation via changes in INPP5K action, total PI content and, more specifically, levels of PI(18:0/20:4), resulting in a compensatory increase in AR action and increased sensitivity to enzalutamide. How AKT and PI phosphatases (like INPP5K) interact in a feedback loop with AR remains to be investigated, offering new therapeutic opportunities as targetable upstream regulators of AR [45, 46].

Importantly, this work demonstrates the increased sensitivity of the CPT1A-deficient cells to enzalutamide *via* activation of AR, consistent with our theory that beta oxidation and AR action are likely reciprocally regulated. These gene/drug interaction findings are further corroborated by the combinatorial treatments of fat burning inhibitors (ranolazine, perhexiline or etomoxir) and enzalutamide in PCa cell models. These combination treatments are robust, non-obvious and have never been tested before as a potential therapy for recurrent or castration-resistance PCa, which is currently incurable. Furthermore, our *in vivo* model with systemic treatments underscores the potential of enhancing the effect of enzalutamide with ranolazine, both FDA approved, providing a pharmacologic paradigm for future clinical investigations. If this combination proves safe/tolerable, the additive effects of adding a lipid oxidation inhibitor on top of an already active anti-androgen agent would be clinically relevant and useful. For example, at the onset of anti-androgen resistance, before radiographic changes occur, CRPC patients could be treated with ranolazine or perhexiline (both approved for human use) in order to restore the sensitivity to enzalutamide and prolong the treatment efficacy.

In conclusion, we show here for the first time that targeting beta-oxidation in combination with androgen blockade results in strong inhibition of PCa growth, by re-sensitizing cells to antiandrogen targeting. Our results reinforce previous studies that AR-targeted therapy maybe insufficient to achieve long term PCa responses in some patients [8] and show that co-targeting the AR axis and fat oxidation may provide additional anti-cancer benefit.

## MATERIALS AND METHODS

### Cell culture and drug treatments

Cell lines (LNCaP and 22Rv1) were obtained from the University of Colorado Cancer Center (UCCC) Tissue Culture Shared Resource (2014) and were authenticated by Single Tandem Repeat analysis. LNCaP MDV-resistant cells were made by growing them in increasing concentrations of MDV ranging from 1.25 to 2.5 uM for 6 months. Cells were passaged in RPMI media containing 5% FBS supplemented with amino acids and Insulin (Hyclone). TRAMPC1 cells (Gift from Dr. Agarwal, 2015, originally from ATCC) were grown in DMEM with 4 mmol/L L-glutamine, 5 μg/mL insulin, 10 nmol/L dihydrotestosterone (DHT), 5% FBS, and 5% Nu Serum (BD Biosciences). Etomoxir-HCl, Ranolazine·2HCL and Perhexiline maleate (all from Sigma-Aldrich, St. Louis, MO) were prepared as a 30, 40 and 15 mM stock solutions, respectively. Enzalutamide (MDV3100, Medivation, Inc), was dissolved as a 20 mM stock in DMSO.

### Immunohistochemistry for CPT1A

Human prostatectomies were obtained from University hospital (UCHealth) using an approved protocol (IRB-00-812). Serial sections of prostatectomies were stained with CPT1A antibody (15184-1-AP, Proteintech, IL) using a 1:1500 dilution and the Benchmark XT I-VIEW DAB detection kit from Ventana (Roche Group). Both CPT1A and H&E stains were done at the University of Colorado Denver Research Histology Shared Resource. A total of 39 prostatectomies (Gleason Score 7 (*n* = 15), 8 (*n* = 12) and 9 (*n* = 12)) were used to assess the CPT1A stain in cancer regions compared to adjacent benign regions. Pathology assessment and final scoring of the stains was done by 2 co-author pathologists (FGL and MSL). Scoring was done by using arbitrary 1, 2 or 3 scores according to intensity of the stains. For each whole prostatectomy, the benign and cancer regions were scored and used as matched values for the analysis.

### Cell viability, proliferation, invasion and clonogenic analysis

Cell viability was analyzed by trypan blue exclusion or by clonogenic assay over 2 weeks. Clonogenic assays were performed by plating 1000 cells/well in 6-well plates and quantitated by ImageJ. Proliferation MTS assays were done by plating 8000 cells/well in 96-well plates and drug treatments lasted 48 hours. Colorimetric analysis was done with CellTiter 96® AQueous Assay from Promega (Madison, WI) according to manufacturer’s instructions. Matrigel invasion assays were performed using Corning® BioCoat™ Tumor Invasion 24-Well Plates according to manufacturer’s protocol.

### CPT1A and INPP5K shRNA transductions

TRCN0000036279 (CPTsh1), TRCN0000036281 (CPTsh2) CPT1A shRNAs and the non-targeting control SHC002 were purchased from the CU Functional Genomics Core. For INPP5K the following shRNA was used: TRCN0000052707 (INPP5K sh). Lentiviral transduction and selection were performed as described [14] or using Xfect Single Shot Transfection (Clonetech).

### CPT1A-CRISPR clones

CPT1A was knocked out by CRISPR /Cas9 technology. Plasmid design and guide RNA sequences are shown in Supplementary Figure 2. LNCaP cells were transduced with a single or combination of the CPT1A CRISPR lentiviral suspension(s) and selected with puromycin (1ug/mL). CPT1A expression was measured in pooled population of cells by RT-PCR. Next, cells were sorted in 96-well plate at the density of 1 cell/well using MoFlo XDP100 Cell sorter by the UCCC Flow Cytometry Core. The single cells clones were expanded and CPT1A knock-out was verified by immunoblotting of cell lysates.

### RNAseq and quantitative RT-PCR and protein interaction network

Attached cells were used for total RNA extraction. The UCCC Genomics and Microarray Core performed cDNA library generation, Illumina HiSeq RNAseq at single read 50 cycles, and generation of FASTQ files. On average, we obtained 36 million reads per sample, and an average mapping of 97.0% to the hg19 reference genome using the tophat/cufflinks workflow as described [47]. Raw RNA-seq data has been deposited into NCBI Gene Expression Omnibus: GSE83547. To determine the differentially expressed genes and pathways between the clones, fragments per kilobase per million mapped reads (FPKM) from each sample were estimated. Data were subjected to bias correction and quartile normalization. For confirmation RTPCR, cDNA was synthesized (Applied Biosystems) and quantified by real-time PCR using SYBR green (Qiagen) detection. Results were normalized to the housekeeping genes B2-macroglobulin or RPL13A mRNA and expressed as arbitrary units of 2−ΔΔCT relative to the control group. List of primers used is shown in Supplementary Table 2. The protein interaction network was generated using the list of common significant genes up and down-regulated (187 up and 232 down) as query for the STRING.v10 software (Search Tool for the Retrieval of Interacting Genes, available at: http://string-db.org/). We used medium confidence (0.4) in the predictions to see all-possible connections. Additionally, we used the STRING database through Cytoscape (http://cytoscape.org/, v3.4) to identify lipid oxidation-relevant interactions of the up- or down-regulated genes.

### Analysis of phospholipids by mass spectrometry

Phospholipid molecular species were extracted from cells and analyzed by liquid chromatography/tandem mass spectrometry as previously described [48]. Data were analyzed using MultiQuant software from AB Sciex (Framingham, MA), and are presented as the ratios between the integrated area of the intensity peak of each analyte and the intensity peak of the corresponding internal standard.

### Immunoblots

Protein extracts 20 μg were separated on a 7.5% SDS-PAGE gel and transferred to nitrocellulose (Invitrogen) as described [49]. Same protein lysates were probed on different blots. All antibodies used are shown in Supplementary Table 3. Band signals were visualized with LICOR system.

### Nude mouse xenograft tumor model

Male athymic nude mice, 4-6 weeks old, were purchased from Charles Rivers Laboratories. Tumor xenografts were generated by injecting human 22Rv1 cells in the flank of nude male mice as described [12]. Approximately 2 × 106 cells were used for each injection. When tumors were palpable the mice were randomized into 4 groups: vehicle, ranolazine (40 mg/kg), enzalutamide (20 mg/Kg) or ranolazine + enzalutamide. These doses were adapted from previous studies where the individual drugs were used safely in mice [50, 51] Treatment was carried out by oral gavage every other day, five days a week, for 21 days. All procedures were carried out under a protocol approved by the Institutional Animal Care and Use Committee of the University of Colorado.

### Statistics

ANOVA tests were used to compare between groups followed by *post hoc* Tukey tests when appropriate. *p* < 0.05 was considered significant. Analysis was carried out with SPSS v23 software (IBM). All data represent mean ±SD. The CalcuSyn software (Biosoft, Ferguson, MO) was used to calculate the combinatorial index of the drug combinations.

## SUPPLEMENTARY MATERIALS FIGURES AND TABLES





## Abbreviations

AR: Androgen receptor
CPT1: Carnitine Palmitoyltransferase I
CRPC: Castration resistant prostate cancer
DHT: Dihydrotestosterone
FFA: Free Fatty Acid
INPP5K: Inositol Polyphosphate-5-Phosphatase K
MDV: MDV3100 or Enzalutamide
MTS: Tetrazolium salt used for colorimetric proliferation assays
PSA: Prostate Specific Antigen
PMS: Perhexiline maleate salt
RRP: radical retropubic prostatectomy
