# Supplementary material for: Lipid catabolism inhibition sensitizes prostate cancer cells to antiandrogen blockade
Source: Oncotarget. 2017 Apr 21;8(34):56051–65. doi: 10.18632/oncotarget.17359 (PMC5593544; doi:10.18632/oncotarget.17359)
Supplement: Supplementary file 1 [file oncotarget-08-56051-s001.pdf]

Lipid catabolism inhibition sensitizes prostate cancer cells to antiandrogen blockade

SUPPLEMENTARY MATERIALS

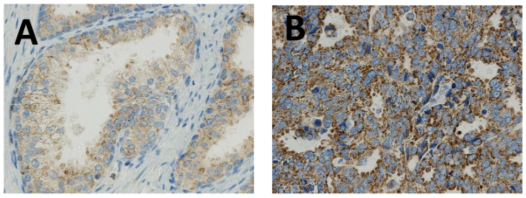

40X images of CPT1A stain in the luminal cells of prostate gland

Data from c-BioPortal

STUDY ABBREVIATION	STUDY NAME	NUM OF CASES ALTERED	PERCENT CASES ALTERED
**NEPC (Trento/Cornell/Broad 2016)**	Neuroendocrine Prostate Cancer (Beltran et al., Nat med 2016)	25	23.40%
**Prostate (SU2C)**	Metastatic Prostate Cancer, SU2C/PCF Dream Team (Robinson et al., Cell 2015)	16	10.70%
**Prostate (MICH)**	Prostate Adenocarcinoma, Metastatic (Michigan, Nature 2012)	3	4.90%
**Prostate (TCGA)**	Prostate Adenocarcinoma (TCGA, Provisional)	7	2.10%
**Prostate (TCGA 2015)**	Prostate Adenocarcinoma (TCGA, Cell 2015)	7	2.10%
**Prostate (Broad/Cornell 2012)**	Prostate Adenocarcinoma (Broad/Cornell, Nat Genet 2012)	2	1.80%

Supplementary Figure 1: Additional images of CPT1a stains at 40X. A) Benign gland. B) Prostate cancer, Gleason pattern 5. Table to the right shows additional information from c-BioPortal on CPT1A gene alterations.

Cloning data for CRISPR editing

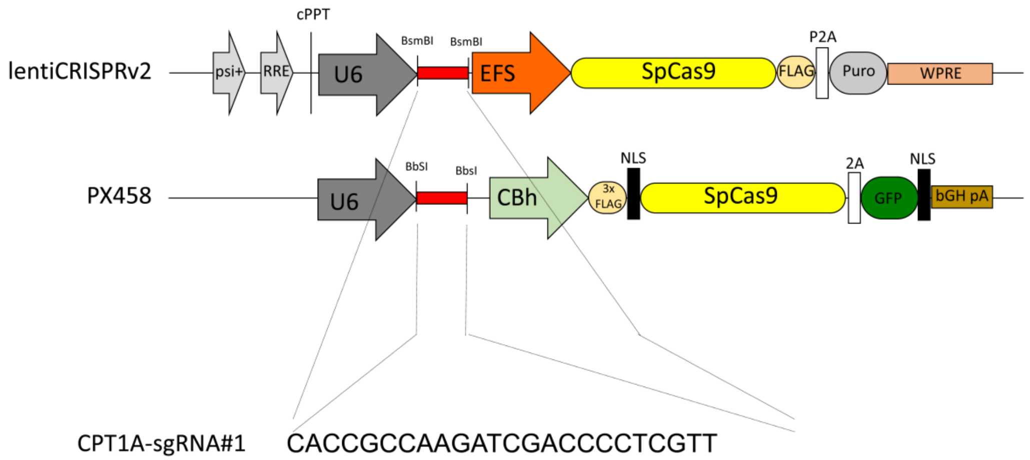

CPT1A-sgRNA#1 CACCGCCAAGATCGACCCCTCGTT
.........CGGTTCTAGCTGGGGAGCAACAAA

CPT1A-sgRNA#2 CACCGACATCGCTGCCTCGCCTGC
CTGTAGCGACGGAGCGGACGCAAA

CPT1A-sgRNA#3 CACCGCAGGCGAGGCAGCGATGTC
CGTCCGCTCCGTCGCTACAGCAAA

NTgA1 CACCGACGGAGGCTAAGCGTCGCAA
CTGCCTCCGATTCGCAGCGTTCAAA

Supplementary Figure 2: Schematic of the CRISPR design to edit CPT1A gene in LNCaP cells. The Functional Genomics Facility at University of Colorado, designed the CRISPR/Cas9 clones. Two different gRNA targeting CPT1A were chosen in combination to produce the most effective knock out: 1) GCCAAGATCGACCCCTCGTT, and 3) GCAGGCGAGGCAGCGATGTC, which were cloned into lentiCRISPRv2 vector (gift from Dr. Feng Zhang lab at the Broad Institute, MIT). One non-targeting control gRNA GACGGAGGCTAAGCGTCGCAA was used for control. Although we designed 2 constructs; the one that uses puromycin as a selection agent (Top) produced the best results and was used in the studies.

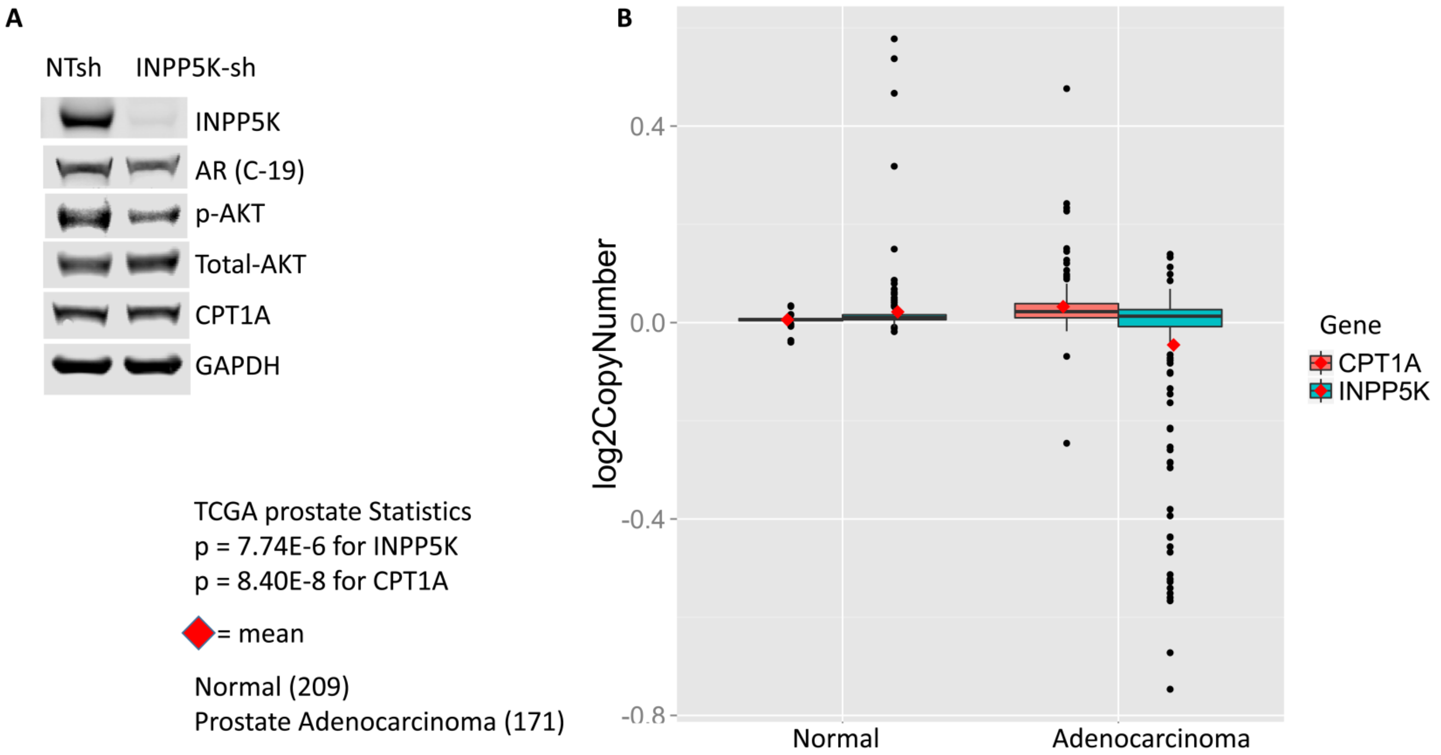

Supplementary Figure 3: **A)** Western blots of LNCaP cell lysates transfected with NT (non-targeting) or INPP5K-shRNA (TRCN0000052707) viral particles. **B)** Gene copy number analysis of CPT1A and INPP5K in TCGA prostate data from Oncomine^{TM} website. Data was downloaded for both genes and plotted as Log2 of the copy number. Line in the boxes represents the median. The red diamond represents the mean for each group.

Supplementary Table 1: CPT1A expression in selected studies from Oncomine™ database**.**

**CPT1A expression from Oncomine**				
**Study data set**	size (N)	p-value	Fold change or correlation	Reference
**1- Metastasis (Primary vs. Metastasis)**				
Liu (Copy number)	58	0.01	1.14	[1]
Grasso (mRNA)	122	2.9×10^{-6}	2.62	[2]
Chandran (mRNA)	31	5.9×10^{-5}	2.65	[3]
Varambally (mRNA)	19	0.025	1.43	[4]
**2- Cancer vs. Normal**				
TCGA Copy number	380	8.4×10^{-8}	1.02	TCGA prostate
Vanaja (mRNA)	40	5.8×10^{-4}	1.09	[5]
**3- Clinical Outcome (dead at 5 years)**				
Setlur (mRNA)	363	1.4×10^{-6}	1.12	[6]
**4- Advanced Gleason Score**				
Luo (mRNA) (6 vs 7)	13	1.5×10^{-4}	1.96	[7]
Setlur (mRNA)	363	4.2×10^{-7}	r = 0.25	[6]
Glinsky (mRNA)	79	0.001	r = 0.35	[8]
Lapointe (mRNA)	58	0.037	r = 0.24	[9]

Supplementary Table 2: Primers		
**name**	**sequence**	**length**
AR (all forms)-F	GAAAGCGACTTCACCGCAC	20
AR (all forms)-R	AAAACATGGTCCCTGGCAGT	21
ARfl-F	ACATCAAGGAACTCGATCGTATCATTGC	28
ARfl-R	TTGGGCACTTGCACAGAGAT	20
ARv7-F	CCATCTTGTCGTCTTCGGAAATGTTATGAAGC	32
ARv7-R	TTTGAATGAGGCAAGTCAGCCTTTCT	26
PSA-F	CGGATGCTGTGAAGGTCATGGAC	23
PSA-R	GGGTCAAGAACTCCTCTGGTTC	21
RPL13A-F	CCTGGAGGAGAAGAGGAAAGAGA	23
RPL13A_R	TTGAGGACCTCTGTGTATTTGTCAA	25

Primers for CPT1A, CLU, ACPP, ETV1, INPP5K, and AZGP1 were purchased from Sigma Aldrich as pre-designed KiCqStart® SYBR® Green Primers.

Supplementary Table 3: Antibodies		
**Antibody**	**Catalogue number**	**Company**
pAKT	4051	Cell Signaling
AKT	9272	Cell Signaling
GAPDH	5174	Cell Signaling
CPT-1A	15184-1-AP	Proteintech
INPP5K	15098-1-AP	Proteintech
AR (C19)	SC-815	Santa Cruz
AR (N20)	SC-816	Santa Cruz
AR-v7	AG10008	Precision
Goat-anti Mouse-HRP	SC-2064	Santa Cruz
Goat-anti Rabbit-HRP	SC-2030	Santa Cruz

Supplementary Table 4: Excel file with all the common genes significantly changed (red=increase/ blue=decreased) in the CPT1AKD clones. KEGG pathway analysis and String Network also included.

For Supplementary Table 4 see in Supplementary Files.

References

1. Liu W, Laitinen S, Khan S, Vihinen M, Kowalski J, Yu G, Chen L, Ewing CM, Eisenberger MA, Carducci MA, Nelson WG, Yegnasubramanian S, Luo J, et al. Copy number analysis indicates monoclonal origin of lethal metastatic prostate cancer. Nat Med. 2009; 15: 559-65.

2. Grasso CS, Wu YM, Robinson DR, Cao X, Dhanasekaran SM, Khan AP, Quist MJ, Jing X, Lonigro RJ, Brenner JC, Asangani IA, Ateeq B, Chun SY, et al. The mutational landscape of lethal castration-resistant prostate cancer. Nature. 2012; 487: 239-43.

3. Chandran UR, Ma C, Dhir R, Bisceglia M, Lyons-Weiler M, Liang W, Michalopoulos G, Becich M, Monzon FA. Gene expression profiles of prostate cancer reveal involvement of multiple molecular pathways in the metastatic process. BMC Cancer. 2007; 7: 64.

4. Varambally S, Yu J, Laxman B, Rhodes DR, Mehra R, Tomlins SA, Shah RB, Chandran U, Monzon FA, Becich MJ, Wei JT, Pienta KJ, Ghosh D, et al. Integrative genomic and proteomic analysis of prostate cancer reveals signatures of metastatic progression. Cancer Cell. 2005; 8: 393-406.

5. Vanaja DK, Cheville JC, Iturria SJ, Young CY. Transcriptional silencing of zinc finger protein 185 identified by expression profiling is associated with prostate cancer progression. Cancer Res. 2003; 63: 3877-82.

6. Setlur SR, Mertz KD, Hoshida Y, Demichelis F, Lupien M, Perner S, Sboner A, Pawitan Y, Andren O, Johnson LA, Tang J, Adami HO, Calza S, et al. Estrogen-dependent signaling in a molecularly distinct subclass of aggressive prostate cancer. J Natl Cancer Inst. 2008; 100: 815-25.

7. Luo JH, Yu YP, Cieply K, Lin F, Deflavia P, Dhir R, Finkelstein S, Michalopoulos G, Becich M. Gene expression analysis of prostate cancers. Mol Carcinog. 2002; 33: 25-35.

8. Glinsky GV, Glinskii AB, Stephenson AJ, Hoffman RM, Gerald WL. Gene expression profiling predicts clinical outcome of prostate cancer. J Clin Invest. 2004; 113: 913-23.

9. Lapointe J, Li C, Higgins JP, van de Rijn M, Bair E, Montgomery K, Ferrari M, Egevad L, Rayford W, Bergerheim U, Ekman P, DeMarzo AM, Tibshirani R, et al. Gene expression profiling identifies clinically relevant subtypes of prostate cancer. Proc Natl Acad Sci U S A. 2004; 101: 811-6.
